# Redesigning a general surgery foundation doctor rota to improve shift equity, staffing levels, doctors’ pay and patient care

**DOI:** 10.1016/j.fhj.2025.100253

**Published:** 2025-04-25

**Authors:** D. Atraszkiewicz

**Affiliations:** Broomfield Hospital, Mid and South Essex NHS Foundation Trust, Court Road, Chelmsford, United Kingdom, CM1 7ET

**Keywords:** Quality improvement, Workplace conditions, Rota design, Patient safety, NHS

## Abstract

Inefficient rota design is a key cause of rota gaps, additional work hours and poor rest among doctors. These are major factors in declining NHS doctor workforce retention rates and compromised patient care. Eighteen improvements were made to the Broomfield Hospital General Surgery foundation doctor rota to improve shift equity, staffing levels, doctors’ pay and patient care. The rota can be used as a template by rota coordinators across NHS trusts. Doctors should be actively involved with improving local rota design

## Highlights

• Efficient rota design is key in preventing rota gaps, additional work hours, poor doctor rest, and compromised patient safety.
• A new rota was created to improve staffing efficiency, doctors' working conditions, and patient care.
• The rota can be used as a template by rota-coordinators across NHS Trusts.
• Doctors should be encouraged to be actively involved with improving local rota design.

## Introduction

Rota gaps and understaffed wards are increasingly common issues among resident doctors.^1^ Subsequently, doctors often work additional hours, sometimes above their grade.^2^^,^^3^ Rota gaps, additional work hours and high staff workloads with little rest are also key factors in the NHS’s low workforce retention rate,^3^ thereby leading to further staff shortages and creating a positive feedback loop. Though low doctor staff levels have been shown to compromise patient safety,^4^ the NHS has an estimated shortfall of 150,000 full-time equivalent workers.^5^

Rota design can also have a significant effect on staff wellbeing. NHS doctors commonly report having a poor work–life balance that negatively impacts their relationships and delays major life events.^6^ Sleep deprivation and doctor ‘burnout’ are linked to twice as many attentional failures^7^ and reduced patient safety.^8^ There is therefore a pertinent need for safe, efficient and effectively designed resident doctor rotas.

Despite this, the 2022/23 general surgery foundation year 1 (FY1) rota at Broomfield Hospital (Mid and South Essex NHS Foundation Trust) had multiple issues. These included: frequent rota gaps with insufficient ward staffing; unequal on-call shift allocations despite doctors receiving identical pay; insufficient rest given between shifts; expectations to work unpaid outside shift hours; sleep patterns being flipped in quick succession; tiresome 56-hour working weeks; limited subspecialty exposure; difficulty booking annual leave; and poor continuity of doctors rostered to work consecutive days.

General surgery is the most populous foundation specialty at Broomfield Hospital; 13 FY1 doctors rotate through the specialty in each 4-month block. Hence, efficient rota design had the potential to make a significant impact. Therefore, in this two-cycle quality improvement (QI) study, a new rota with multiple improvements was designed and implemented with the aim of improving minimum ward staffing, doctor wellbeing and patient care.

## Method

A ten-question baseline survey was created using Google® Forms and Survey Monkey® tools to evaluate the previous rota (Appendix 1). Data were collected between September 2022 and January 2023 from FY1 doctors with general surgery placements in block 1 (August 2022 to December 2022) and block 2 (December 2022 to April 2023). Additionally, as a doctor on general surgery and urology within block 1, the rota was routinely analysed during shifts to identify real-time causes of rota inefficiencies and inequalities. Further areas for improvement were identified from discussions within Junior Doctor Forum (JDF) meetings.

National and local regulations governing rota compliance were researched via internet searches. Additionally, a meeting was arranged with the Human Resources (HR) department to better understand the rota constraints due to the European Working Time Directive (EWTD). Various rota iterations were designed on Microsoft® Excel spreadsheets until a compliant rota with the maximum number of achievable improvements (18) was created. The new rota was presented to senior general surgery consultants, who approved the improvements made. The rota was then reviewed by the HR department to ensure that all required compliance regulations were met and to adjust doctors’ work schedules accordingly. Further meetings with hospital management, rota coordinators and the JDF escalated implementation of the rota and also allowed improvements to be shared with other departments within the hospital trust.

The new rota was implemented for FY1 doctors with general surgery placements in block 3 (April 2023 to August 2023). A ten-question post-implementation survey was distributed between May 2023 and June 2023 to gather feedback on the new rota (Appendix 2).

## Results

Analysis of the previous rota showed that a key cause of unequal rota allocations among doctors was the manual addition of ‘theatre’ weeks, ‘vascular’ weeks and sSelf-development time’ (SDEV) onto the rota by a rota coordinator. This caused variation in patterns of shifts worked and also led to some doctors not receiving their full contractual allocation of certain shift types. The new rota (Appendix 3) therefore incorporates all these shift types into a master rota – which each doctor rotates through – thereby ensuring that shift allocations across the 13-week rota cycle are equal between doctors, including subspecialty exposure.

Additionally, numerous changes were made to improve staff work–life balance. The maximum allocation of night and long day on-call shifts per doctor reduced from 14 to 11 and these are more equitably allocated (range reduced from seven to four). Likewise, the maximum allocation of short day on-call shifts reduced from 12 to 11. Doctors with higher allocations of night on-call shifts work fewer day on-call shifts, and vice versa. Every doctor now has a full allocation of vascular NWDs and SDEV days as per their contract. Doctors on urology / general surgery mixed placements now have an equal placement split. Finally, a new shift is created starting at 7:30am, ensuring that doctors are paid fairly for time spent preparing morning patient lists and ward notes. Annualised additional hours pay therefore increased from £5,325.84 to £5,509.50 (3.4%). Though not an outcome measure, ward rounds were anecdotally reported to run smoother and more efficiently.

**Fig. 1:**
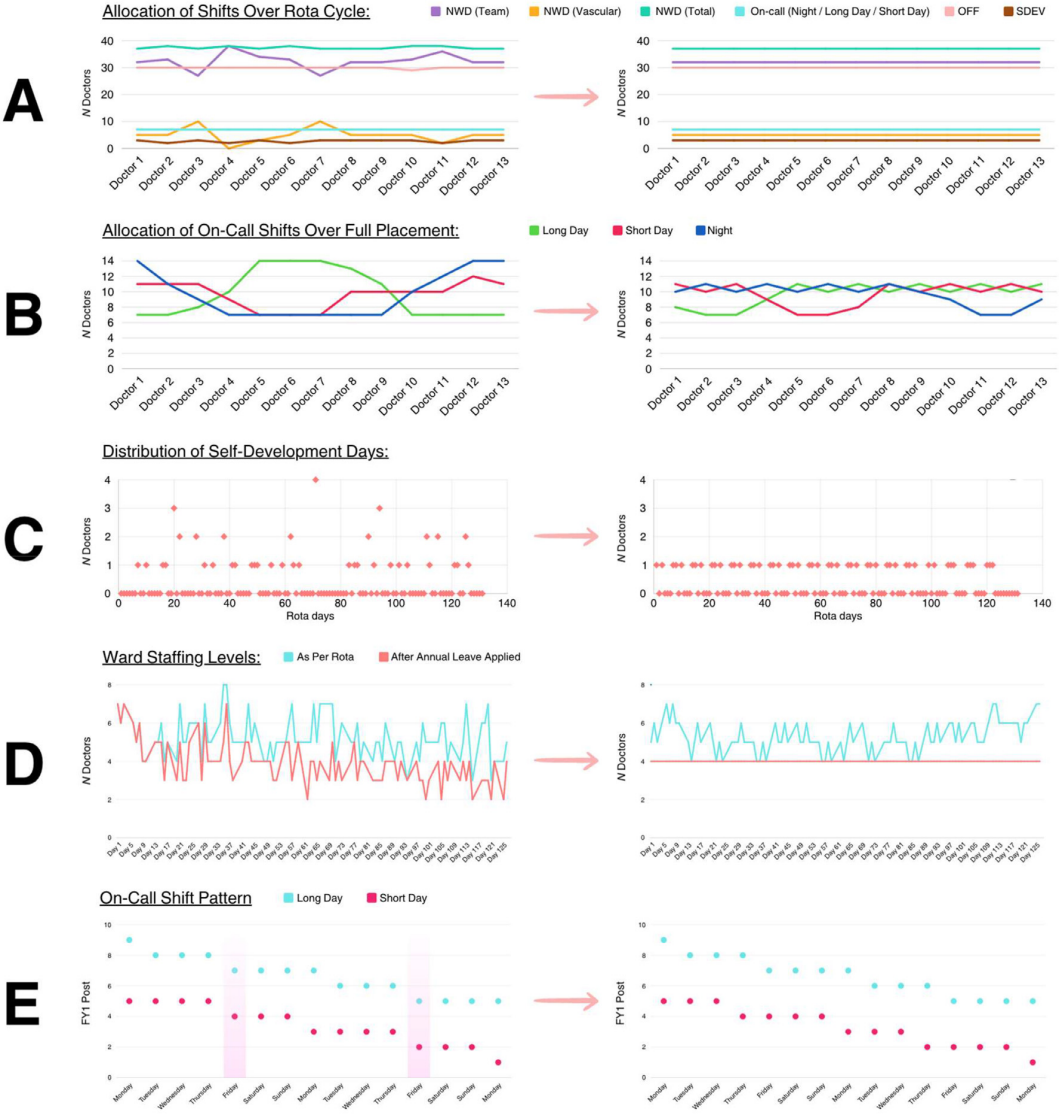
Graphs showing rota improvements made. (Left: previous rota; Right: new rota). (**A**) There is now an equal allocation of all shift types, self-development days and ‘off' days across the 13-week rota cycle for all doctors. Purple: normal working day (NWD) shifts with specialty; orange: NWD with vascular team; teal: NWD total; blue: on-call shifts; pink: oOff’ shifts; brown: self-development days. (**B**) All on-call shift types are more fairly allocated across the full placement with range reduced from seven to four. Each doctor works a lesser allowance of one on-call type. Green: long day on-call; red: short day on-call; dark blue: night. (**C**) There’s now a maximum of one FY1 doctor at any point on a self-development (SDEV) day, thereby maximising ward staffing levels. (**D**) Minimum ward staffing is increased from two doctors to four. Blue: NWD ward doctor levels as per master rota; pink: NWD ward doctor levels after annual leave (and days off in lieu due from bank holidays etc.) applied. (**E**) Previously on Fridays, doctors rotated on both ‘long’ and ‘short' on-call shifts, resulting in poor continuity of patient care. Doctors now rotate on different days, ensuring that one doctor is always familiar with the patient list. Blue: long day on-call; pink: short day on-call.

The new rota also has no rota gaps. This was achieved via a number of changes. First, SDEV allocations are more efficiently distributed across the full placement length. This reduced the maximum number of doctors with SDEV on any given day from four to one (Fig. 1). Additionally, night shift allocations now rotate between subspecialties, thereby increasing minimum ward doctor levels from two to four. A core trainee is therefore also no longer required to adjust the rota weekly to fill gaps, and can instead be deployed to support clinical duties. Subsequently, no locum staff are required to fill rota gaps (excluding unforeseen absence due to staff sickness).

Rest from clinical duties following on-call shifts is significantly increased. This was achieved by placing SDEV and ‘off’ days before and after night shifts. Likewise, ‘theatre’ and ‘vascular’ weeks (with objectively lower clinical workloads) are placed after night shifts. Previously, day on-call shifts inefficiently followed night shifts. Night shifts are now separated into two stints on the rota, further increasing rest time to adjust sleep patterns. On-call shifts are also better distributed on the rota, reducing the longest working week from 56 h to 50 h.

Continuity of patient care is also improved by increasing the continuity of doctors working NWD shifts on surgical wards through the week. Additionally, doctors on short day and long day on-call shifts now rotate on different days (rather than both rotating on Fridays previously), further ensuring continuity of patient care.

In addition to the above rota improvements, a spreadsheet was also created to expedite annual leave (AL) booking while ensuring minimum staff levels. This can be distributed 8 weeks prior to the start of placements and doctors can request AL dates on a first come, first served basis. Additionally, a week spent with the urology department for all doctors was suggested to improve subspecialty exposure. Support for such a week was high (85% of FY1 doctors agreed); however, this could not be implemented due to inter-departmental funding restraints.

**Fig. 2:**
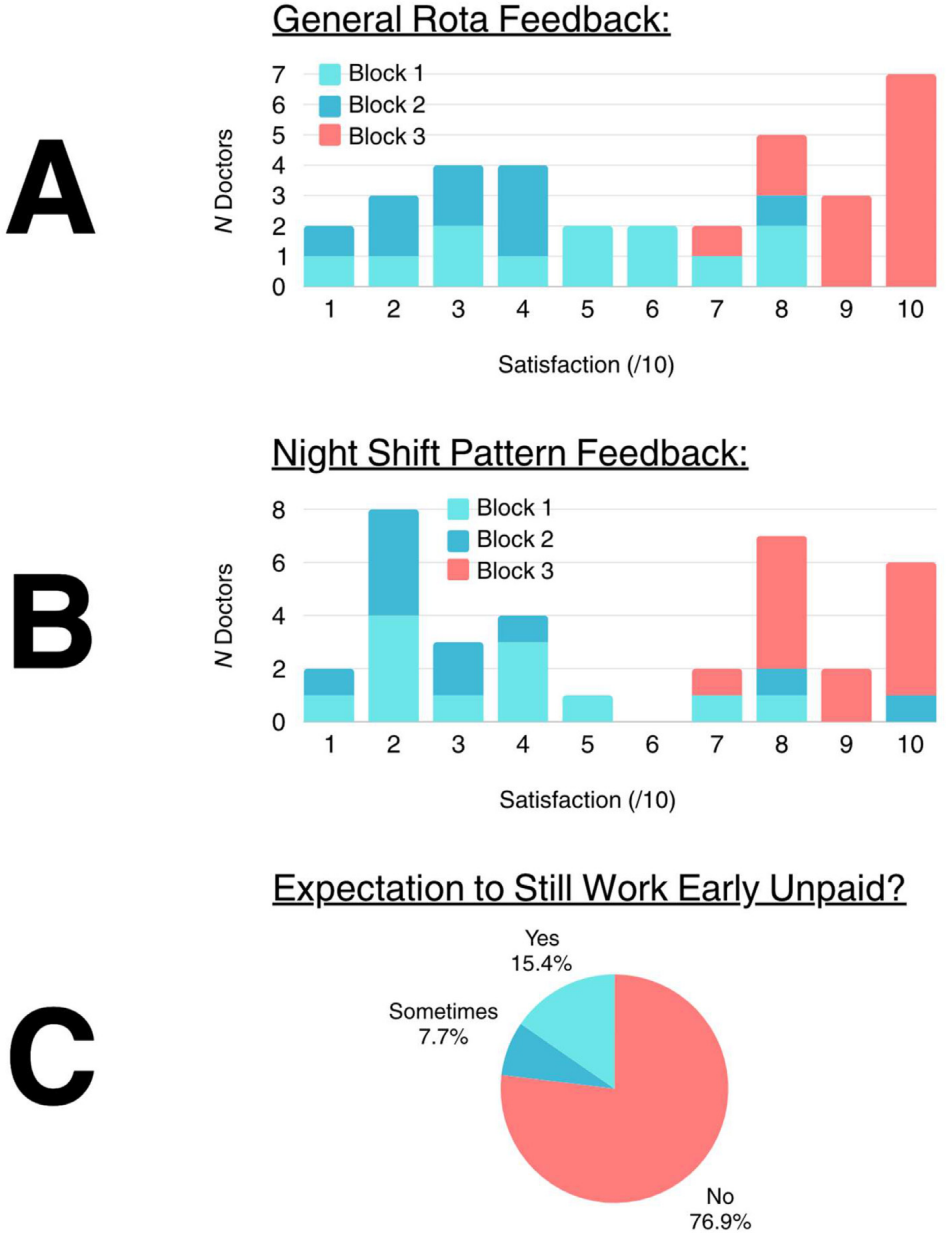
Charts showing survey responses. Total responses: 34 (89.5%). New rota implemented in block 3. (**A**) Average satisfaction with the rota increased from 4.24 out of 10 (SD 2.18) to 9.23 (SD 0.97). Blue: block 1; light blue: block 2; pink: block 3. (**B**) Average satisfaction with night shift patterns increased from 3.48 out of 10 (SD 2.28) to 8.85 (SD 1.03). Blue: block 1; light blue: block 2; pink: block 3. (**C**) 77% of doctors were no longer working early unpaid to prepare patient lists and ward notes. Pink: no; blue: sometimes; light blue: yes.

There were 21 (84%) responses to the baseline survey and 13 (100%) responses to the post-implementation survey (Fig. 2). Average rota satisfaction increased from 4.24 out of 10 (SD 2.18) to 9.23 (SD 0.97). Likewise, average satisfaction with night shift patterns increased from 3.48 (SD 2.28) to 8.85 (SD 1.03). The new rota was preferred by 92% of doctors. 77% of doctors reported no longer performing unpaid work (an increase from 0%).

These multiple rota improvements can be implemented by rota coordinators across NHS trusts. The 13-week master rota (Appendix 4) ensures full shift equality for 13 doctors over the rota cycle. For cohorts with more or fewer doctors, the rota can be amended by respectively adding, or removing, a week of normal working day (NWD) shifts (eg week 7). If other NHS trusts do not offer ‘theatre’ or vVascular’ weeks on general surgery placements, weeks 3 and/or 9 can be replaced with NWD shifts.

## Discussion

Rota design is a key focus of numerous NHS staff wellbeing case studies, including self-rostering systems, rota compliance and flexible working arrangements.^9^ However, in contrast to such studies, this QI study relied on very few contextual factors: HR and management teams were crucial in authorising and approving the new rota, while the rota team were required to distribute this to the doctor staff. The remainder of the study was conceived, designed, conducted and analysed independently by one FY1 doctor. Therefore, this study can be easily reproduced by other NHS trusts to implement similar rota improvements more widely.

A systematic review found that healthcare self-rostering systems improved rostering efficiency, staff empowerment, and staff work–life balance.^10^ However, compared to fixed rostering, self-rostering resulted in reduced fairness of rota allocations, implementation challenges, more shift change requests and increased overtime.^10^ There is therefore a continued need to improve traditional fixed rostering methods. Doctors should be encouraged to actively work with rota coordinators to amend rota issues, a stance also advocated for by the British Medical Association.^11^

## Limitations

A limitation of this study was that the current resident doctor contract states all work schedules should be ‘averaged over the shorter of the rota cycle, placement length, or 26 weeks’.^12^ It therefore was not possible to achieve equal shift allocations over the full placement length (17–18 weeks) as the rota cycle (13 weeks) is not a divisible factor. Additionally, financial savings due to reduced expenditure on locum staff could not be assessed as these data were not available from the department. The baseline and post-implementation surveys were also not formally validated.

## Conclusions

Eighteen improvements were made to the general surgery FY1 rota at Broomfield Hospital, resulting in: equal shift allocations across the master rota; greater rest between on-call shifts; fairer pay for additional hours worked (3.4% pay increase); and safer doctor staffing (minimum ward doctor levels increased from 2 to 4). The rota can be used as a template by rota coordinators across NHS trusts to improve wider patient care. Doctors are encouraged to be actively involved with rota design.

## Ethics approval and consent to participate

Not applicable.

## Funding

This research did not receive any specific grant from funding agencies in the public, commercial or not-for-profit sectors.

## Data availability statement

All data are available within the article and supplementary material.

## CRediT authorship contribution statement

**D. Atraszkiewicz:** Writing – review & editing, Writing – original draft, Visualization, Project administration, Methodology, Investigation, Formal analysis, Data curation, Conceptualization.

## Declaration of competing interest

The author declares that they have no known competing financial interests or personal relationships that could have appeared to influence the work reported in this paper.

## Appendix Supplementary materials

Image, application 1
