# Supplementary material for: Redesigning a general surgery foundation doctor rota to improve shift equity, staffing levels, doctors’ pay and patient care
Source: Future Healthc J. 2025 Apr 25;12(2):100253. doi: 10.1016/j.fhj.2025.100253 (PMC12137149; doi:10.1016/j.fhj.2025.100253)
Supplement: Supplementary file 1 [file mmc1.pdf]

APPENDICES

Appendix 1: Screenshot of baseline feedback survey — data collected between Sept 2022 to Jan 2023.

General Surgery FY1 Rota 22/23 Feedback (Block 1)

* 1. On a scale of 1-10, how happy are you with the current General Surgery FY1 rota?

* 2. Have you had / Do you have any issues with the current rota?

* 3. Do you have any suggestions on how to improve the rota? / Is there anything you would like in a new rota?

* 4. How many days off do you feel is needed to switch sleep patterns before Night shifts?

* 5. On a scale of 1-10, how happy are you with the current set up for Night shifts?
(i.e. 1 OFF, 3 Nights, 2 OFF, 3 NWD, 2 OFF, 4 Nights)?

* 6. What time do you arrive onto the wards for a NWD shift?

Date / Time

Time: hh : mm — AM/PM: -

7. On average, what is the minimum number of FY1s you feel is needed to cover NWD shifts? (i.e. across both Colorectal and UGI in total)

* 8. Have you had any issues with booking off annual leave? (If so, please explain)

* 9. Have you exception reported? (If so, please state the number of times)

10. Have you had any problems with Exception Reporting? (If so, please explain)

Done

Appendix 2: Screenshot of post-implementation feedback survey — data collected between May 2023 to June 2023.

General Surgery FY1 Rota Feedback

Please take a minute to complete the questions below - thank you!

* Indicates required question

Q1: How happy are you with the new General Surgery FY1 rota?

	1	2	3	4	5	6	7	8	9	10	
Not Happy	○	○	○	○	○	○	○	○	○	○	Couldn't be happier

Q2: How happy are you with the number of 'rest days' given before and after night shifts? (i.e. Off days and SDEV allocations)

	1	2	3	4	5	6	7	8	9	10	
Not Happy	○	○	○	○	○	○	○	○	○	○	Couldn't be happier

Q3. Do you prefer the new pattern of Night shifts?(Previously this was: 1 OFF, 3 Nights, 2 OFF, 3 NWD, 2 OFF, 4 Nights, 3 OFF, 4 On-calls) *

- ○ Yes
- ○ No
- ○ Not sure

Q4: How happy are you with the number of days of rest before and after On-call shifts?

	1	2	3	4	5	6	7	8	9	10	
Not happy	○	○	○	○	○	○	○	○	○	○	Couldn't be happier

Q5: How happy are you with the current NWD ward cover levels?

	1	2	3	4	5	6	7	8	9	10	
Not happy	○	○	○	○	○	○	○	○	○	○	Couldn't be happier

Q6. Are you still expected to arrive early to prepare the lists on a NWD shift? (i.e. when you're not on a 7:30AM shift week) *

Your answer

Q7. Have you had any issues with booking annual leave? (If so, please explain) *

Your answer

Q8. Have you needed to exception report on Gen Surg? (If so, please state the number of times) *

Your answer

Q9. Do you have any further suggestions on how to improve the rota? Or are there any other rota comments or concerns you'd like to escalate?

Your answer

Q10: How would you feel about Urology becoming a one week rotation that all General Surgery FY1 doctors rated through? (With the aim of all FY1 doctors being more familiar with managing Urology patients when they're doing on-call shifts.) *

- ○ Bad idea
- ○ Good idea
- ○ I don't know

Appendix 3: Table showing the new General Surgery Foundation Year 1 rota.

			Colorectal	Upper GI	Colorectal	Upper GI	Colorectal	Upper GI	Colorectal	Upper GI	Colorectal	Upper GI	Colorectal	UGI/Urology	Urology/UGI
			Doctor 1	Doctor 2	Doctor 3	Doctor 4	Doctor 5	Doctor 6	Doctor 7	Doctor 8	Doctor 9	Doctor 10	Doctor 11	Doctor 12	Doctor 13
Week 1	MONDAY	n/a	n/a	n/a	n/a	n/a	n/a	n/a	n/a	n/a	n/a	n/a	n/a	n/a	n/a
	TUESDAY	n/a	n/a	n/a	n/a	n/a	n/a	n/a	n/a	n/a	n/a	n/a	n/a	n/a	n/a
	WEDNESDAY	3/8/22	SDEV	0800-1700	OFF	0800-2030	0800-1700	0800-1700	0800-1900	OFF	0730-1700	0800-1700	0800-1700	0800-1700	2000-0830
	THURSDAY	4/8/22	OFF	0800-1700	0800-1700	0800-2030	OFF	0800-1700	0800-1700	0800-1900	0730-1700	0800-1700	0800-1700	0800-1700	2000-0830
	FRIDAY	5/8/22	2000-0830	0800-1700	0800-1700	SDEV	0800-2030	0800-1700	OFF	0800-1900	0730-1700	0800-1700	0800-1700	0800-1700	OFF
	SATURDAY	6/8/22	2000-0830	OFF	OFF	OFF	0800-2030	OFF	OFF	0800-1700	OFF	OFF	OFF	OFF	OFF
	SUNDAY	7/8/22	2000-0830	OFF	OFF	OFF	0800-2030	OFF	OFF	0800-1700	OFF	OFF	OFF	OFF	OFF
Week 2	MONDAY	8/8/22	OFF	2000-0830	0800-1700	0800-1700	0800-2030	0800-1700	0800-1700	OFF	0800-1900	0800-1700	0730-1700	0800-1700	0800-1700
	TUESDAY	9/8/22	OFF	2000-0830	0800-1700	0800-1700	OFF	0800-2030	0800-1700	SDEV	0800-1900	0800-1700	0730-1700	0800-1700	0800-1700
	WEDNESDAY	10/8/22	0800-1700	2000-0830	SDEV	0800-1700	OFF	0800-2030	0800-1700	0800-1700	0800-1900	OFF	0730-1700	0800-1700	0800-1700
	THURSDAY	11/8/22	0800-1700	2000-0830	OFF	0800-1700	0800-1700	0800-2030	OFF	0800-1700	0800-1700	0800-1900	0730-1700	0800-1700	0800-1700
	FRIDAY	12/8/22	0800-1700	OFF	2000-0830	0800-1700	0800-1700	SDEV	0800-2030	0800-1700	OFF	0800-1900	0730-1700	0800-1700	0800-1700
	SATURDAY	13/8/22	OFF	OFF	2000-0830	OFF	OFF	OFF	0800-2030	OFF	OFF	0800-1700	OFF	OFF	OFF
	SUNDAY	14/8/22	OFF	OFF	2000-0830	OFF	OFF	OFF	0800-2030	OFF	OFF	0800-1700	OFF	OFF	OFF
Week 3	MONDAY	15/8/22	0800-1700	0800-1700	OFF	2000-0830	0800-1700	0800-1700	0800-2030	0800-1700	0800-1700	OFF	0800-1900	0800-1700	0730-1700
	TUESDAY	16/8/22	0800-1700	0800-1700	OFF	2000-0830	0800-1700	0800-1700	OFF	0800-2030	0800-1700	SDEV	0800-1900	0800-1700	0730-1700
	WEDNESDAY	17/8/22	0800-1700	0800-1700	0800-1700	2000-0830	SDEV	0800-1700	OFF	0800-2030	0800-1700	0800-1700	0800-1900	OFF	0730-1700
	THURSDAY	18/8/22	0800-1700	0800-1700	0800-1700	2000-0830	OFF	0800-1700	0800-1700	0800-2030	OFF	0800-1700	0800-1700	0800-1900	0730-1700
	FRIDAY	19/8/22	0800-1700	0800-1700	0800-1700	OFF	2000-0830	0800-1700	0800-1700	SDEV	0800-2030	0800-1700	OFF	0800-1900	0730-1700
	SATURDAY	20/8/22	OFF	OFF	OFF	OFF	2000-0830	OFF	OFF	OFF	0800-2030	OFF	OFF	0800-1700	OFF
	SUNDAY	21/8/22	OFF	OFF	OFF	OFF	2000-0830	OFF	OFF	OFF	0800-2030	OFF	OFF	0800-1700	OFF
Week 4	MONDAY	22/8/22	0800-1700	0730-1700	0800-1700	0800-1700	OFF	2000-0830	0800-1700	0800-1700	0800-2030	0800-1700	0800-1700	OFF	0800-1900
	TUESDAY	23/8/22	0800-1700	0730-1700	0800-1700	0800-1700	OFF	2000-0830	0800-1700	0800-1700	OFF	0800-2030	0800-1700	SDEV	0800-1900
	WEDNESDAY	24/8/22	OFF	0730-1700	0800-1700	0800-1700	0800-1700	2000-0830	SDEV	0800-1700	OFF	0800-2030	0800-1700	0800-1700	0800-1900
	THURSDAY	25/8/22	0800-1900	0730-1700	0800-1700	0800-1700	0800-1700	2000-0830	OFF	0800-1700	0800-1700	0800-2030	OFF	0800-1700	0800-1700
	FRIDAY	26/8/22	0800-1900	0730-1700	0800-1700	0800-1700	0800-1700	OFF	2000-0830	0800-1700	0800-1700	SDEV	0800-2030	0800-1700	OFF
	SATURDAY	27/8/22	0800-1700	OFF	OFF	OFF	OFF	OFF	2000-0830	OFF	OFF	OFF	0800-2030	OFF	OFF
	SUNDAY	28/8/22	0800-1700	OFF	OFF	OFF	OFF	OFF	2000-0830	OFF	OFF	OFF	0800-2030	OFF	OFF
Week 5	MONDAY	29/8/22	OFF	0800-1900	0800-1700	0730-1700	0800-1700	0800-1700	OFF	2000-0830	0800-1700	0800-1700	0800-2030	0800-1700	0800-1700
	TUESDAY	30/8/22	SDEV	0800-1900	0800-1700	0730-1700	0800-1700	0800-1700	OFF	2000-0830	0800-1700	0800-1700	OFF	0800-2030	0800-1700
	WEDNESDAY	31/8/22	0800-1700	0800-1900	OFF	0730-1700	0800-1700	0800-1700	0800-1700	2000-0830	SDEV	0800-1700	OFF	0800-2030	0800-1700
	THURSDAY	1/9/22	0800-1700	0800-1700	0800-1900	0730-1700	0800-1700	0800-1700	0800-1700	2000-0830	OFF	0800-1700	0800-1700	0800-2030	OFF
	FRIDAY	2/9/22	0800-1700	OFF	0800-1900	0730-1700	0800-1700	0800-1700	0800-1700	OFF	2000-0830	0800-1700	0800-1700	SDEV	0800-2030
	SATURDAY	3/9/22	OFF	OFF	0800-1700	OFF	OFF	OFF	OFF	OFF	2000-0830	OFF	OFF	OFF	0800-2030
	SUNDAY	4/9/22	OFF	OFF	0800-1700	OFF	OFF	OFF	OFF	OFF	2000-0830	OFF	OFF	OFF	0800-2030
Week 6	MONDAY	5/9/22	0800-1700	0800-1700	OFF	0800-1900	0800-1700	0730-1700	0800-1700	0800-1700	OFF	2000-0830	0800-1700	0800-1700	0800-2030
	TUESDAY	6/9/22	0800-2030	0800-1700	SDEV	0800-1900	0800-1700	0730-1700	0800-1700	0800-1700	OFF	2000-0830	0800-1700	0800-1700	OFF
	WEDNESDAY	7/9/22	0800-2030	0800-1700	0800-1700	0800-1900	OFF	0730-1700	0800-1700	0800-1700	0800-1700	2000-0830	SDEV	0800-1700	OFF
	THURSDAY	8/9/22	0800-2030	OFF	0800-1700	0800-1700	0800-1900	0730-1700	0800-1700	0800-1700	0800-1700	2000-0830	OFF	0800-1700	0800-1700
	FRIDAY	9/9/22	SDEV	0800-2030	0800-1700	OFF	0800-1900	0730-1700	0800-1700	0800-1700	0800-1700	OFF	2000-0830	0800-1700	0800-1700
	SATURDAY	10/9/22	OFF	0800-2030	OFF	OFF	0800-1700	OFF	OFF	OFF	OFF	OFF	2000-0830	OFF	OFF
	SUNDAY	11/9/22	OFF	0800-2030	OFF	OFF	0800-1700	OFF	OFF	OFF	OFF	OFF	2000-0830	OFF	OFF
Week 7	MONDAY	12/9/22	0800-1700	0800-2030	0800-1700	0800-1700	OFF	0800-1900	0800-1700	0730-1700	0800-1700	0800-1700	OFF	2000-0830	0800-1700
	TUESDAY	13/9/22	0800-1700	OFF	0800-2030	0800-1700	SDEV	0800-1900	0800-1700	0730-1700	0800-1700	0800-1700	OFF	2000-0830	0800-1700
	WEDNESDAY	14/9/22	0800-1700	OFF	0800-2030	0800-1700	0800-1700	0800-1900	OFF	0730-1700	0800-1700	0800-1700	0800-1700	2000-0830	SDEV
	THURSDAY	15/9/22	0800-1700	0800-1700	0800-2030	OFF	0800-1700	0800-1700	0800-1900	0730-1700	0800-1700	0800-1700	0800-1700	2000-0830	OFF
	FRIDAY	16/9/22	0800-1700	0800-1700	SDEV	0800-2030	0800-1700	OFF	0800-1900	0730-1700	0800-1700	0800-1700	0800-1700	OFF	2000-0830
	SATURDAY	17/9/22	OFF	OFF	OFF	0800-2030	OFF	OFF	0800-1700	OFF	OFF	OFF	OFF	OFF	2000-0830
	SUNDAY	18/9/22	OFF	OFF	OFF	0800-2030	OFF	OFF	0800-1700	OFF	OFF	OFF	OFF	OFF	2000-0830
Week 8	MONDAY	19/9/22	2000-0830	0800-1700	0800-1700	0800-2030	0800-1700	0800-1700	OFF	0800-1900	0800-1700	0730-1700	0800-1700	0800-1700	OFF
	TUESDAY	20/9/22	2000-0830	0800-1700	0800-1700	OFF	0800-2030	0800-1700	SDEV	0800-1900	0800-1700	0730-1700	0800-1700	0800-1700	OFF
	WEDNESDAY	21/9/22	2000-0830	SDEV	0800-1700	OFF	0800-2030	0800-1700	0800-1700	0800-1900	OFF	0730-1700	0800-1700	0800-1700	0800-1700
	THURSDAY	22/9/22	2000-0830	OFF	0800-1700	0800-1700	0800-2030	OFF	0800-1700	0800-1700	0800-1900	0730-1700	0800-1700	0800-1700	0800-1700
	FRIDAY	23/9/22	OFF	2000-0830	0800-1700	0800-1700	SDEV	0800-2030	0800-1700	OFF	0800-1900	0730-1700	0800-1700	0800-1700	0800-1700
	SATURDAY	24/9/22	OFF	2000-0830	OFF	OFF	OFF	0800-2030	OFF	OFF	0800-1700	OFF	OFF	OFF	OFF
	SUNDAY	25/9/22	OFF	2000-0830	OFF	OFF	OFF	0800-2030	OFF	OFF	0800-1700	OFF	OFF	OFF	OFF
Week 9	MONDAY	26/9/22	0800-1700	OFF	2000-0830	0800-1700	0800-1700	0800-2030	0800-1700	0800-1700	OFF	0800-1900	0800-1700	0730-1700	0800-1700
	TUESDAY	27/9/22	0800-1700	OFF	2000-0830	0800-1700	0800-1700	OFF	0800-2030	0800-1700	SDEV	0800-1900	0800-1700	0730-1700	0800-1700
	WEDNESDAY	28/9/22	0800-1700	0800-1700	2000-0830	SDEV	0800-1700	OFF	0800-2030	0800-1700	0800-1700	0800-1900	OFF	0730-1700	0800-1700
	THURSDAY	29/9/22	0800-1700	0800-1700	2000-0830	OFF	0800-1700	0800-1700	0800-2030	OFF	0800-1700	0800-1700	0800-1900	0730-1700	0800-1700
	FRIDAY	30/9/22	0800-1700	0800-1700	OFF	2000-0830	0800-1700	0800-1700	SDEV	0800-2030	0800-1700	OFF	0800-1900	0730-1700	0800-1700
	SATURDAY	1/10/22	OFF	OFF	OFF	2000-0830	OFF	OFF	OFF	0800-2030	OFF	OFF	0800-1700	OFF	OFF
	SUNDAY	2/10/22	OFF	OFF	OFF	2000-0830	OFF	OFF	OFF	0800-2030	OFF	OFF	0800-1700	OFF	OFF
Week 10	MONDAY	3/10/22	0730-1700	0800-1700	0800-1700	OFF	2000-0830	0800-1700	0800-1700	0800-2030	0800-1700	0800-1700	OFF	0800-1900	0800-1700
	TUESDAY	4/10/22	0730-1700	0800-1700	0800-1700	OFF	2000-0830	0800-1700	0800-1700	OFF	0800-2030	0800-1700	SDEV	0800-1900	0800-1700
	WEDNESDAY	5/10/22	0730-1700	0800-1700	0800-1700	0800-1700	2000-0830	SDEV	0800-1700	OFF	0800-2030	0800-1700	0800-1700	0800-1900	OFF
	THURSDAY	6/10/22	0730-1700	0800-1700	0800-1700	0800-1700	2000-0830	OFF	0800-1700	0800-1700	0800-2030	OFF	0800-1700	0800-1700	0800-1900
	FRIDAY	7/10/22	0730-1700	0800-1700	0800-1700	0800-1700	OFF	2000-0830	0800-1700	0800-1700	SDEV	0800-2030	0800-1700	OFF	0800-1900
	SATURDAY	8/10/22	OFF	OFF	OFF	OFF	OFF	2000-0830	OFF	OFF	OFF	0800-2030	OFF	OFF	0800-1700
	SUNDAY	9/10/22	OFF	OFF	OFF	OFF	OFF	2000-0830	OFF	OFF	OFF	0800-2030	OFF	OFF	0800-1700
Week 11	MONDAY	10/10/22	0800-1900	0800-1700	0730-1700	0800-1700	0800-1700	OFF	2000-0830	0800-1700	0800-1700	0800-2030	0800-1700	0800-1700	OFF
	TUESDAY	11/10/22	0800-1900	0800-1700	0730-1700	0800-1700	0800-1700	OFF	2000-0830	0800-1700	0800-1700	OFF	0800-2030	0800-1700	SDEV
	WEDNESDAY	12/10/22	0800-1900	OFF	0730-1700	0800-1700	0800-1700	0800-1700	2000-0830	SDEV	0800-1700	OFF	0800-2030	0800-1700	0800-1700
	THURSDAY	13/10/22	0800-1700	0800-1900	0730-1700	0800-1700	0800-1700	0800-1700	2000-0830	OFF	0800-1700	0800-1700	0800-2030	OFF	0800-1700
	FRIDAY	14/10/22	OFF	0800-1900	0730-1700	0800-1700	0800-1700	0800-1700	OFF	2000-0830	0800-1700	0800-1700	SDEV	0800-2030	0800-1700
	SATURDAY	15/10/22	OFF	0800-1700	OFF	OFF	OFF	OFF	OFF	2000-0830	OFF	OFF	OFF	0800-2030	OFF
	SUNDAY	16/10/22	OFF	0800-1700	OFF	OFF	OFF	OFF	OFF	2000-0830	OFF	OFF	OFF	0800-2030	OFF
Week 12	MONDAY	17/10/22	0800-1700	OFF	0800-1900	0800-1700	0730-1700	0800-1700	0800-1700	OFF	2000-0830	0800-1700	0800-1700	0800-2030	0800-1700
	TUESDAY	18/10/22	0800-1700	SDEV	0800-1900	0800-1700	0730-1700	0800-1700	0800-1700	OFF	2000-0830	0800-1700	0800-1700	OFF	0800-2030
	WEDNESDAY	19/10/22	0800-1700	0800-1700	0800-1900	OFF	0730-1700	0800-1700	0800-1700	0800-1700	2000-0830	SDEV	0800-1700	OFF	0800-2030
	THURSDAY	20/10/22	OFF	0800-1700	0800-1700	0800-1900	0730-1700	0800-1700	0800-1700	0800-1700	2000-0830	OFF	0800-1700	0800-1700	0800-2030
	FRIDAY	21/10/22	0800-2030	0800-1700	OFF	0800-1900	0730-1700	0800-1700	0800-1700	0800-1700	OFF	2000-0830	0800-1700	0800-1700	SDEV
	SATURDAY	22/10/22	0800-2030	OFF	OFF	0800-1700	OFF	OFF	OFF	OFF	OFF	2000-0830	OFF	OFF	OFF
	SUNDAY	23/10/22	0800-2030	OFF	OFF	0800-1700	OFF	OFF	OFF	OFF	OFF	2000-0830	OFF	OFF	OFF
Week 13	MONDAY	24/10/22	0800-2030	0800-1700	0800-1700	OFF	0800-1900	0800-1700	0730-1700	0800-1700	0800-1700	OFF	2000-0830	0800-1700	0800-1700
	TUESDAY	25/10/22	OFF	0800-2030	0800-1700	SDEV	0800-1900	0800-1700	0730-1700	0800-1700	0800-1700	OFF	2000-0830	0800-1700	0800-1700
	WEDNESDAY	26/10/22	OFF	0800-2030	0800-1700	0800-1700	0800-1900	OFF	0730-1700	0800-1700	0800-1700	0800-1700	2000-0830	SDEV	0800-1700
	THURSDAY	27/10/22	0800-1700	0800-2030	OFF	0800-1700	0800-1700	0800-1900	0730-1700	0800-1700	0800-1700	0800-1700	2000-0830	OFF	0800-1700
	FRIDAY	28/10/22	0800-1700	SDEV	0800-2030	0800-1700	OFF	0800-1900	0730-1700	0800-1700	0800-1700	0800-1700	OFF	2000-0830	0800-1700
	SATURDAY	29/10/22	OFF	OFF	0800-2030	OFF	OFF	0800-1700	OFF	OFF	OFF	OFF	OFF	2000-0830	OFF
	SUNDAY	30/10/22	OFF	OFF	0800-2030	OFF	OFF	0800-1700	OFF	OFF	OFF	OFF	OFF	2000-0830	OFF
Week 14	MONDAY	31/10/22	0800-1700	0800-1700	0800-2030	0800-1700	0800-1700	OFF	0800-1900	0800-1700	0730-1700	0800-1700	0800-1700	OFF	2000-0830
	TUESDAY	1/11/22	0800-1700	0800-1700	OFF	0800-2030	0800-1700	SDEV	0800-1900	0800-1700	0730-1700	0800-1700	0800-1700	OFF	2000-0830
	WEDNESDAY	2/11/22	SDEV	0800-1700	OFF	0800-2030	0800-1700	0800-1700	0800-1900	OFF	0730-1700	0800-1700	0800-1700	0800-1700	2000-0830
	THURSDAY	3/11/22	OFF	0800-1700	0800-1700	0800-2030	OFF	0800-1700	0800-1700	0800-1900	0730-1700	0800-1700	0800-1700	0800-1700	2000-0830
	FRIDAY	4/11/22	2000-0830	0800-1700	0800-1700	SDEV	0800-2030	0800-1700	OFF	0800-1900	0730-1700	0800-1700	0800-1700	0800-1700	OFF
	SATURDAY	5/11/22	2000-0830	OFF	OFF	OFF	0800-2030	OFF	OFF	0800-1700	OFF	OFF	OFF	OFF	OFF
	SUNDAY	6/11/22	2000-0830	OFF	OFF	OFF	0800-2030	OFF	OFF	0800-1700	OFF	OFF	OFF	OFF	OFF
Week 15	MONDAY	7/11/22	OFF	2000-0830	0800-1700	0800-1700	0800-2030	0800-1700	0800-1700	OFF	0800-1900	0800-1700	0730-1700	0800-1700	0800-1700
	TUESDAY	8/11/22	OFF	2000-0830	0800-1700	0800-1700	OFF	0800-2030	0800-1700	0800-1700	0800-1900	0800-1700	0730-1700	0800-1700	0800-1700
	WEDNESDAY	9/11/22	0800-1700	2000-0830	SDEV	0800-1700	OFF	0800-2030	0800-1700	0800-1700	0800-1900	OFF	0730-1700	0800-1700	0800-1700
	THURSDAY	10/11/22	0800-1700	2000-0830	OFF	0800-1700	0800-1700	0800-2030	OFF	0800-1700	0800-1700	0800-1900	0730-1700	0800-1700	0800-1700
	FRIDAY	11/11/22	0800-1700	OFF	2000-0830	0800-1700	0800-1700	SDEV	0800-2030	0800-1700	OFF	0800-1900	0730-1700	0800-1700	0800-1700
	SATURDAY	12/11/22	OFF	OFF	2000-0830	OFF	OFF	OFF	0800-2030	OFF	OFF	0800-1700	OFF	OFF	OFF
	SUNDAY	13/11/22	OFF	OFF	2000-0830	OFF	OFF	OFF	0800-2030	OFF	OFF	0800-1700	OFF	OFF	OFF
Week 16	MONDAY	14/11/22	0800-1700	0800-1700	OFF	2000-0830	0800-1700	0800-1700	0800-2030	0800-1700	0800-1700	OFF	0800-1900	0800-1700	0730-1700
	TUESDAY	15/11/22	0800-1700	0800-1700	OFF	2000-0830	0800-1700	0800-1700	OFF	0800-2030	0800-1700	0800-1700	0800-1900	0800-1700	0730-1700
	WEDNESDAY	16/11/22	0800-1700	0800-1700	0800-1700	2000-0830	SDEV	0800-1700	OFF	0800-2030	0800-1700	0800-1700	0800-1900	OFF	0730-1700
	THURSDAY	17/11/22	0800-1700	0800-1700	0800-1700	2000-0830	OFF	0800-1700	0800-1700	0800-2030	OFF	0800-1700	SDEV	0800-1900	0730-1700
	FRIDAY	18/11/22	0800-1700	0800-1700	0800-1700	OFF	2000-0830	0800-1700	0800-1700	SDEV	0800-2030	0800-1700	OFF	0800-1900	0730-1700
	SATURDAY	19/11/22	OFF	OFF	OFF	OFF	2000-0830	OFF	OFF	OFF	0800-2030	OFF	OFF	0800-1700	OFF
	SUNDAY	20/11/22	OFF	OFF	OFF	OFF	2000-0830	OFF	OFF	OFF	0800-2030	OFF	OFF	0800-1700	OFF
Week 17	MONDAY	21/11/22	0800-1700	0730-1700	0800-1700	0800-1700	OFF	2000-0830	0800-1700	0800-1700	0800-2030	0800-1700	0800-1700	OFF	0800-1900
	TUESDAY	22/11/22	0800-1700	0730-1700	0800-1700	0800-1700	OFF	2000-0830	0800-1700	0800-1700	OFF	0800-2030	0800-1700	0800-1700	0800-1900
	WEDNESDAY	23/11/22	OFF	0730-1700	0800-1700	0800-1700	0800-1700	2000-0830	SDEV	0800-1700	OFF	0800-2030	0800-1700	0800-1700	0800-1900
	THURSDAY	24/11/22	0800-1900	0730-1700	0800-1700	0800-1700	0800-1700	2000-0830	OFF	0800-1700	0800-1700	0800-2030	OFF	0800-1700	SDEV
	FRIDAY	25/11/22	0800-1900	0730-1700	0800-1700	0800-1700	0800-1700	OFF	2000-0830	0800-1700	0800-1700	SDEV	0800-2030	0800-1700	OFF
	SATURDAY	26/11/22	0800-1700	OFF	OFF	OFF	OFF	OFF	2000-0830	OFF	OFF	OFF	0800-2030	OFF	OFF
	SUNDAY	27/11/22	0800-1700	OFF	OFF	OFF	OFF	OFF	2000-0830	OFF	OFF	OFF	0800-2030	OFF	OFF
Week 18	MONDAY	28/11/22	OFF	0800-1900	0800-1700	0730-1700	0800-1700	0800-1700	OFF	2000-0830	0800-1700	0800-1700	0800-2030	0800-1700	0800-1700
	TUESDAY	29/11/22	0800-1700	0800-1900	0800-1700	0730-1700	0800-1700	0800-1700	OFF	2000-0830	0800-1700	0800-1700	OFF	0800-2030	0800-1700
	WEDNESDAY	30/11/22	0800-1700	0800-1900	OFF	0730-1700	0800-1700	0800-1700	0800-1700	2000-0830	SDEV	0800-1700	OFF	0800-2030	0800-1700
	THURSDAY	1/12/22	0800-1700	SDEV	0800-1900	0730-1700	0800-1700	0800-1700	0800-1700	2000-0830	OFF	0800-1700	0800-1700	0800-2030	OFF
	FRIDAY	2/12/22	0800-1700	OFF	0800-1900	0730-1700	0800-1700	0800-1700	0800-1700	OFF	2000-0830	0800-1700	0800-1700	SDEV	0800-2030
	SATURDAY	3/12/22	OFF	OFF	0800-1700	OFF	OFF	OFF	OFF	OFF	2000-0830	OFF	OFF	OFF	0800-2030
	SUNDAY	4/12/22	OFF	OFF	0800-1700	OFF	OFF	OFF	OFF	OFF	2000-0830	OFF	OFF	OFF	0800-2030
Week 19	MONDAY	5/12/22	0800-1700	0800-1700	OFF	0800-1900	0800-1700	0730-1700	0800-1700	0800-1700	OFF	2000-0830	0800-1700	0800-1700	0800-2030
	TUESDAY	6/12/22	0800-2030	0800-1700	0800-1700	0800-1900	0800-1700	0730-1700	0800-1700	0800-1700	OFF	2000-0830	0800-1700	0800-1700	OFF

Key:
Normal Working Day (General Surgery)
Normal Working Day (Urology)
List Preparation Week (Gen Surgery)
List Preparation Week (Urology)
Long Day On-Call
Short Day On-Call
Night
OFF
Self-Development (SDEV)
Weekend (Off)
Vascular Week (NWD)
Theatre Week (NWD)

Appendix 4: Table showing the master rota for 13-week rota cycle. This can serve as a template for rota-coordinators in other NHS trusts.

Master Rota							
	**Monday**	**Tuesday**	**Wednesday**	**Thursday**	**Friday**	**Saturday**	**Sunday**
**Week 1**			**SDEV**	OFF	2000-0830	2000-0830	2000-0830
**Week 2**	OFF	OFF	0800-1700	0800-1700	0800-1700	OFF	OFF
**Week 3**	0800-1700	0800-1700	0800-1700	0800-1700	0800-1700	OFF	OFF
**Week 4**	0800-1700	0800-1700	OFF	0800-1900	0800-1900	0800-1700	0800-1700
**Week 5**	OFF	**SDEV**	0800-1700	0800-1700	0800-1700	OFF	OFF
**Week 6**	0800-1700	0800-2030	0800-2030	0800-2030	**SDEV**	OFF	OFF
**Week 7**	0800-1700	0800-1700	0800-1700	0800-1700	0800-1700	OFF	OFF
**Week 8**	2000-0830	2000-0830	2000-0830	2000-0830	OFF	OFF	OFF
**Week 9**	0800-1700	0800-1700	0800-1700	0800-1700	0800-1700	OFF	OFF
**Week 10**	0730-1700	0730-1700	0730-1700	0730-1700	0730-1700	OFF	OFF
**Week 11**	0800-1900	0800-1900	0800-1900	0800-1700	OFF	OFF	OFF
**Week 12**	0800-1700	0800-1700	0800-1700	OFF	0800-2030	0800-2030	0800-2030
**Week 13**	0800-2030	OFF	OFF	0800-1700	0800-1700	OFF	OFF
**Week 14**	0800-1700	0800-1700	-	-	-	-	-

**Key:**
Normal Working Day (NWD)
List Preparation Week
Long Day On-Call
Short Day On-Call
Night
OFF
**Self-Development (SDEV)**
Vascular Week (NWD)
Theatre Week (NWD)
